# Effect of exogenous thermophilic biocontrol agent inoculum on the high temperature chicken manure composting

**DOI:** 10.3389/fmicb.2024.1484047

**Published:** 2024-11-15

**Authors:** Zuojun Liu, Qiang Yin, Yong Fang, Xueping Zhang, Wensheng Xia, Zhentong Jiao, Tao Song, Heyan Wan, Ting Guo

**Affiliations:** ^1^Agriculture Mechanization and Engineering Research Institute, Anhui Academy of Agricultural Sciences, Hefei, China; ^2^Jiangsu Academy of Agricultural Sciences, Nanjing, China

**Keywords:** chicken manure, composting, microbial community, thermophilic inoculant, metabolite

## Abstract

**Introduction:**

Aerobic composting is an effective method for utilizing chicken manure. However, its low carbon to nitrogen (C/N) ratio leads to slow heating and short high-temperature phases, which reduce composting efficiency and product quality.

**Methods:**

To address this issue, splinted mushroom cultivation residues were added to adjust the C/N ratio, and exogenous thermophilic composting strains were introduced to increase composting temperature. This study analyzed the relationship between physicochemical metabolites and microbial community structure during high-temperature chicken manure composting.

**Results and discussion:**

Based on metagenomic and physicochemical analyses, results showed that the exogenous microbial agents extended the thermophilic phase by three-times, reduced the heating phase duration by 75%, and increased nitrogen, phosphorus, potassium, and soluble organic carbon contents by 3.61, 21.63, 7.21, and 39.03%, respectively. Genes associated with amino acid metabolism were significantly enriched during the heating phase, while genes involved in the tricarboxylic acid cycle were more active in the thermophilic phase. During the thermophilic phase, bacterial diversity and richness decreased compared to the heating and cooling phases. Functional microbes such as *Bacillus*, *Caldicoprobacter*, and *Virgibacillus* showed a positive correlation with the key differential metabolites. While *Actinomadura*, *Saccharomonospora*, *Paenibacillus*, and *Aneurinibacillus* displayed an opposite correlation. Further experiments demonstrated that the increased temperature during the thermophilic phase triggered the upregulation of oleic acid metabolism and piperidine metabolism pathways in functional microorganisms, leading to the production of heat stabilizers and protective agents like oleic acid, gallic acid, and 2-piperidone. This phenomenon helped maintain microbial viability during the thermophilic phase and improved composting efficiency.

## 1 Introduction

Approximately 3.8 billion tons of livestock manure are produced annually in China, with chicken manure representing about 15% of this total (Guo et al., 2020). Chicken manure is rich in various nutrients, including nitrogen (N), phosphorus (P), potassium (K) and humus, which enhances its potential application in agriculture (Chen et al., 2020). However, chicken manure cannot be used directly, as improper treatment can lead to increased emissions of malodorous gases, greenhouse gases, pathogens, and heavy metals (Awasthi et al., 2020). Aerobic composting, has been recognized as an effective treatment method for manure utilization, successfully transforming chicken manure into humus through microbial action. Research indicates that composted chicken manure can improve soil quality, reduce crop reliance on chemical fertilizers, and enhance crop yield (Antonangelo et al., 2021). Nonetheless, the high nitrogen content, low carbon to nitrogen (C/N) ratio, and high moisture content of chicken manure present challenges when initiating the composting process.

Aerobic composting consists of mesophilic, thermophilic, and mature stages, each dominated by specific microbial populations. The mesophilic stage is characterized by a rise in ambient temperature to approximately 50°C. During this phase, functional microbes initiate the decomposition of organic matter while releasing heat, thereby increasing the temperature of the composting. Once the temperature exceeds 50°C, the process transitions into the thermophilic stage, during which complex organic materials are further degraded and temperatures continue to rise. As the substrate available for thermophilic microorganisms decreases and their activity declines, the compost temperature gradually decreases, entering the mature phase.

In the mesophilic stage, common functional microbes such as *Bacillus* and *Pseudomonas*, thrive by utilizing easily degradable sugars and proteins as nutrients. The growth and reproduction of these microbes develop conditions favorable for the subsequent thermophilic stage, resulting in a gradual increase in compost temperature. If the C/N ratio during this period does not meet the growth requirements of the microbes, it can hinder their reproduction, causing a slow temperature rise or even stagnation, which ultimately impedes the composting process. To address this issue, rice husks were added to chicken manure to adjust the C/N ratio prior to composting. This adjustment not only enhanced composting efficiency but also led to a significant reduction in NH_3_ and CH_4_ emissions by 56.12 and 62.24%, respectively (Li et al., 2023).

During the thermophilic phase, thermophilic microbes emerge as the dominant bacterial group, while the relative abundance of mesophilic microbes diminishes. Certain thermophilic microorganisms, including *Bacillus* and *Streptomyces*, flourish at elevated temperatures, efficiently decomposing complex organic matter, such as cellulose, into simpler compounds. This process not only increases compost temperature but also promotes the maturation of the compost (Xu et al., 2023). The elevated temperatures effectively eradicate harmful substances, including pathogens and weed seeds, thereby enhancing the quality of chicken manure composting. Furthermore, maintaining optimal temperatures during composting can reduce the composting cycle, improve the biotransformation rate, and yield high-quality end products (Sun P. et al., 2022). Extending the thermophilic phase serves as an effective strategy for enhancing composting efficiency (Li et al., 2020). In the maturation phase, as temperatures begin to decline, the abundance and activity of thermophilic microorganisms decrease, allowing mesophilic microorganisms and certain saprophytic organisms to reactivate and restore bacterial diversity. During this stage, these microorganisms continue to decompose the remaining refractory organic matter, leading to the formation of humus, which enhances soil structure and fertility, further stabilizing the compost.

Microorganisms, acting as internal drivers of biological transformation, play an indispensable role in the composting process (Liu et al., 2018; Sun S. et al., 2022). To enhance the efficiency of chicken manure composting, the exogenous addition of microbial inoculum (*B. subtilis*, *E. hormaechei*, and *T. reesei*), has been shown to elevate the temperature during the thermophilic phase by 3–7°C (Li et al., 2024). Furthermore, inoculating with the fungus *Phanerochaete chrysosporium* can significantly enhance the degradation of cellulose and lignin, thereby promoting the humification process within composting (Chen et al., 2019). The dynamic changes in microbial communities and their metabolic functions govern the transitions between different periods during composting. Numerous researchers are exploring how microbial community structures evolve through various composting stages to analyze the driving mechanisms of these microorganisms. In studies examining the co-composting of rice bran and chicken manure, it was found that Clostridiaceae dominated during the mesophilic phase, whereas Bacteriaceae became predominant in the thermophilic phase (Peng et al., 2018). There exists a cohesive relationship between microbial succession and metabolic function (Wu et al., 2022). In the composting of deceased chickens, populations of Proteobacteria, Bacteroidetes, and Deinococcus-Thermus gradually increased with extended composting duration. Additionally, amino acid and carbohydrate metabolism, as identified in the Kyoto Encyclopedia of Genes and Genomes pathway for bacterial function, emerged as the primary microbial metabolic pathways influencing final degradation efficiency (Lin et al., 2022). However, the slow warming process, short duration of high temperatures, and excessive composting time (1–4 months) associated with chicken manure result in suboptimal compost products (Cao et al., 2020; Deng et al., 2020), severely constraining the effective utilization of chicken manure resources.

In China, there is an abundance of mushroom residues, the majority of which are not being effectively utilized. As a cost-effective and efficient supplement in chicken manure composting, mushroom residues are rich in residual organic matter, including proteins and lignocellulose, while also exhibiting low moisture content and a high C/N ratio (Nishimura et al., 2021). Co-composting agricultural waste alongside livestock manure presents an optimized approach to overcome challenges associated with the solitary decomposition of chicken manure and mushroom residues (Wu et al., 2022). However, research focused on the utilization of *Agaricus bisporus* residues in chicken manure co-composting, particularly with detailed process monitoring, remains insufficient. Additionally, a comprehensive understanding of the impact of temperature on material metabolism and microbial community structure during chicken manure composting is still needed.

To effectively utilize agricultural waste resources, a mixture of eight biocontrol agents, including *B. licheniformis*, *B. megaterium*, *B. subtilis*, *B. amyloliquefaciens*, *S. cerevisiae*, *C. funkei*, *Caldicoprobacter* sp., and *Virgibacillus* sp., was inoculated during the initial period of co-composting chicken manure and *A. bisporus* residues. This study investigated the impact of inoculation on the dynamics of physicochemical parameters, the evolution of the bacterial community, and metabolic functions. The interactions among temperature, bacterial community, and metabolic under temperature-driven conditions were evaluated. The results suggest a promising composting method for producing high-quality organic fertilizers.

## 2 Materials and methods

### 2.1 Experimental materials

A mixture of chicken manure and *A. bisporus* residue was prepared at a ratio of 3:2 (fresh weight) and served as the primary raw material. Samples were collected from a chicken farm and a mushroom plantation located in the Wanzhi District, Wuhu City, Anhui Province, China. The biocontrol agents used included *B. licheniformis*, *B. megaterium*, *B. subtilis*, *B. amyloliquefaciens*, *C. funkei*, *Caldicoprobacter* sp., *Virgibacillus* sp., and *S. cerevisiae*. The strains were sourced from chicken manure and decayed wood. Bacterial strains were cultured in Luria Bertani medium at 37°C, while fungal strains were cultured in Yeast Extract Peptone Dextrose medium at 30°C. The colony-forming units (CFU) of the eight strains were determined using the colony counting method. After equal mixing of these strains, a mixed biocontrol agent inoculum with a CFU of 1 × 10^8^ was prepared.

### 2.2 Experimental design

Each compost heap contained approximately 300 kg of chicken manure and 200 kg of mushroom residues, resulting in a C/N ratio of 25. The moisture content was adjusted to ~ approximately 50% by adding water. The heaps were shaped trapezoidally, with a bottom width of 2 m, a top width of 1 m, and a height of 1.5 m. A biocontrol agent noculation rate of 1.0% (v/v) was applied. Throughout the composting process, temperature and pH were measured at five points every 24 h at 8:00 am at a depth of 40 cm, and ambient temperature was also recorded. Aeration was facilitated by mechanical turning every 24 h at 9:00 am. Samples were collected at the initial fermentation, mesophilic, thermophilic, and mature phases (days 0, 2, 16, 32). Five points were randomly arranged at a depth of 40 cm, and approximately 200 g of sample was collected at each point. The samples from each phases were mixed, placed in coolers, and promptly transported to the laboratory, where they were stored at −20°C for further analysis.

### 2.3 Physiochemical properties test

Total organic matter (TOM), dissolved organic carbon (DOC), pH, N, P, and K were determined according to the Chinese Ministry of Agriculture’s industry standard (NY/T 1116-2014). The total organic carbon (TOC) measurement adhered to the methodology outlined in the protocol established by the Chinese Ministry of Environmental Protection (HJ 615-2011). For each indicator, three parallel samples were analyzed.

### 2.4 Untargeted analysis of metabolites

Samples weighing 100 mg were resuspended in 400 μL of an extraction solvent (acetonitrile:methanol, 1:1) by vortexing, and the resulting extract supernatant was concentrated in a vacuum concentrator. The metabolites were then redissolved in a different extraction solvent (acetonitrile:water, 1:1) and separated using ultra-high-performance liquid chromatography (UPLC) equipped with a UPLC column (ACQUITY UPLC BEH Amide 1.7 μm, 2.1 × 100 mm, Waters, Milford, MA). The mobile phase A consisted of 25 mM ammonium acetate and 25 mM ammonium hydroxide in water, while mobile phase B consisted of 100% acetonitrile. The following gradient program was used: 95% B, 0.5 min; 95–65% B, 0.5–7 min; 65–40% B, 7–8 min; 40% B, 9 min; 40–95% B, 9–9.1 min; and 95% B, 12 min. The flow rate was maintained at 0.5 mL/min and the injection volume was 2 μL, with sample storage at 4°C in an autosampler. The following mass spectrometry (MS) conditions were set: ion spray voltage (IonSpray Voltage Floating), at 5500 V; ion gas temperature, at 650°C; ion gas pressure, at 60 psi; curtain gas, at 30 psi; and declustering potential, at 60 V.

### 2.5 Exploration of compost microbial communities

DNA samples were extracted using a PowerSoil DNA Isolation Kit (MoBio Laboratories, Carlsbad, CA, United States), in accordance with the manufacturer’s instructions. The purity and quality of the extracted genomic DNA were assessed using 1% agarose gels and a NanoDrop2000 spectrophotometer (Thermo Fisher Scientific, Inc., United States). The V3-4 hypervariable region of the bacterial 16S rRNA gene was amplified with the primers 338F (5’-ACTCCTACGGGAGGCAGCAG-3′) and 806R (5’-GGACTACNNGGGTATCTAAT-3′). Polymerase chain reaction (PCR) was performed on a Mastercycler Gradient (Eppendorf, Germany) utilizing 25 μL reaction volumes that consisted of 12.5 μL 2 × Taq PCR MasterMix, 3 μL BSA (2 ng/μL), 1 μL Forward Primer (5 μM), 1 μL Reverse Primer (5 μM), 2 μL template DNA, and 5.5 μL ddH_2_O. Cycler conditions included an initial denaturation at 95°C for 5 min, followed by 28 cycles at 95°C for 45 s, 55°C for 50 s, and 72°C for 45 s, concluding with a final extension at 72°C for 10 min. The PCR products were then purified using an Agencourt AMPure XP Kit (Beckman Coulter, Inc., United States). Subsequently, high-throughput sequencing of the PCR products was conducted by Allwegene Company (Beijing). Raw sequencing data were screened, and sequences shorter than 120 bp, with a low quality score (≤ 20), containing ambiguous bases, or not matching precisely to primer sequences and barcode tags were excluded. The remaining sequences were sorted based on sample-specific barcode sequences. Qualified reads were clustered into operational taxonomic units (OTUs) at a similarity threshold of 97% using the Uparse algorithm via Vsearch (v2.7.1) software. Taxonomic classification of all sequences was performed using the BLAST tool against the Silva138 database. Quantitative Insights Into Microbial Ecology (QIIME v1.8.0) was utilized to generate rarefaction curves and to calculate richness and diversity indices based on the OTU data. To analyze the community membership and structure across different samples, heatmaps of the top 20 OTUs were generated using Mothur. Based on taxonomic annotations and relative abundance results, statistical analyses were conducted using R (v3.6.0) for bar plot diagram generation. Clustering analyses and Principal Component Analysis (PCA) were performed in R (v3.6.0) to examine the similarity between different samples, utilizing the OTU data from each. Evolutionary distances between microbial communities from each sample were calculated using the Bray-Curtis algorithm, resulting in an Unweighted Pair Group Method with Arithmetic Mean (UPGMA) clustering tree that illustrates the dissimilarity (1-similarity) among the various samples. A Newick-formatted tree file was generated through this analysis.

### 2.6 Thermal stability analysis

The compost inoculant was divided into 5 mL portions, each containing a CFU of 1 × 10^8^. Oleic acid, gallic acid, and 2-piperidinone were individually added to each sample to achieve a final concentration of 3 mg/mL. In the control group, an equivalent volume of sterile water was added. Each sample was prepared independently.

### 2.7 Statistical analyses

Statistical analyses (*p* < 0.05) were conducted using SPSS 20.0 to analyze the relationship between material transformation and microbial community composting. Origin software was used for cartographic analysis.

## 3 Results

### 3.1 Variations in temperature, pH, and major nutrients during composting

**Figure 1:**
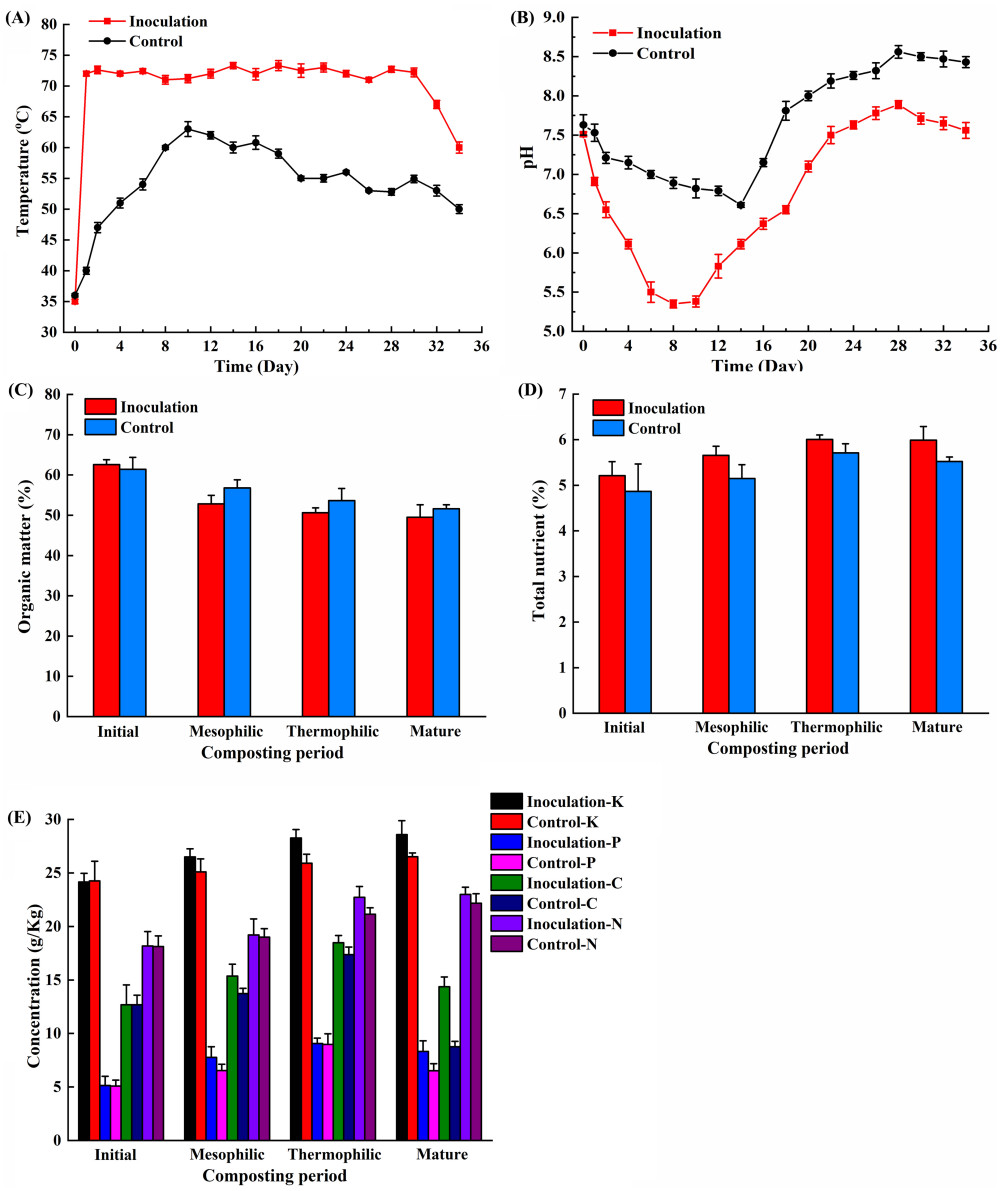
Changes in physicochemical properties of chicken manure composting. **(A)** Temperature; **(B)** pH; **(C)** Organic matter; **(D)** Total nutrient; **(E)** Potassium, phosphorus, nitrogen and soluble organic carbon. The data presented are the average values from triplicate technical repeats of measurements. The error bars represent the standard deviations of the means.

The dynamic temperature during the composting process was monitored. Following the composting period, the temperatures in both the control and treatment groups began to rise. In the inoculation group, which included an exogenous microbial agent, the temperature reached 72°C within 24 h, entering the thermophilic phase and maintaining that temperature for 30 days. In contrast, the control group exhibited a more gradual temperature increase, reaching 60°C on the 8th day and sustaining it for 10 days. The peak temperatures recorded for the inoculation and control groups were 74°C and 63°C, respectively (Figure 1A). The pH values for both groups displayed a trend of initial decline, followed by an increase, and a final decrease (Figure 1B). During the initial phase, the pH levels in the inoculation and control groups dropped rapidly from 7.51 and 7.63 to 5.35 and 6.61 on the 8th and 14th days, respectively. As the fermentation progressed, the pH levels began to increase. The highest stable pH values observed were 7.89 for the inoculation group and 8.56 for the control group. After the composting process, the organic matter content in the control group was 51.61%, whereas the inoculation group recorded a value of 49.52% (*p* < 0.05) (Figure 1C). The total nutrient content increased throughout the composting process. After composting, the total nutrient content was 5.99% for the inoculation group and 5.52% for the control group (*p* < 0.05) (Figure 1D). After fermentation, the contents of nitrogen, phosphorus, and potassium in the inoculation group were 23.00 g/kg, 8.32 g/kg, and 28.58 g/kg, compared to 22.17 g/kg, 6.52 g/kg, and 26.52 g/kg in the control group (*p* < 0.05) (Figure 1E). Both groups exhibited a pattern of soluble organic carbon that initially increased, followed by a decrease. With the addition of exogenous microbial inoculants, the soluble organic carbon content for the inoculation and control groups was measured at 14.37 g/kg and 8.76 g/kg, respectively (*p* < 0.05) (Figure 1E).

### 3.2 Differential metabolites and metabolic pathways analysis

**Figure 2:**
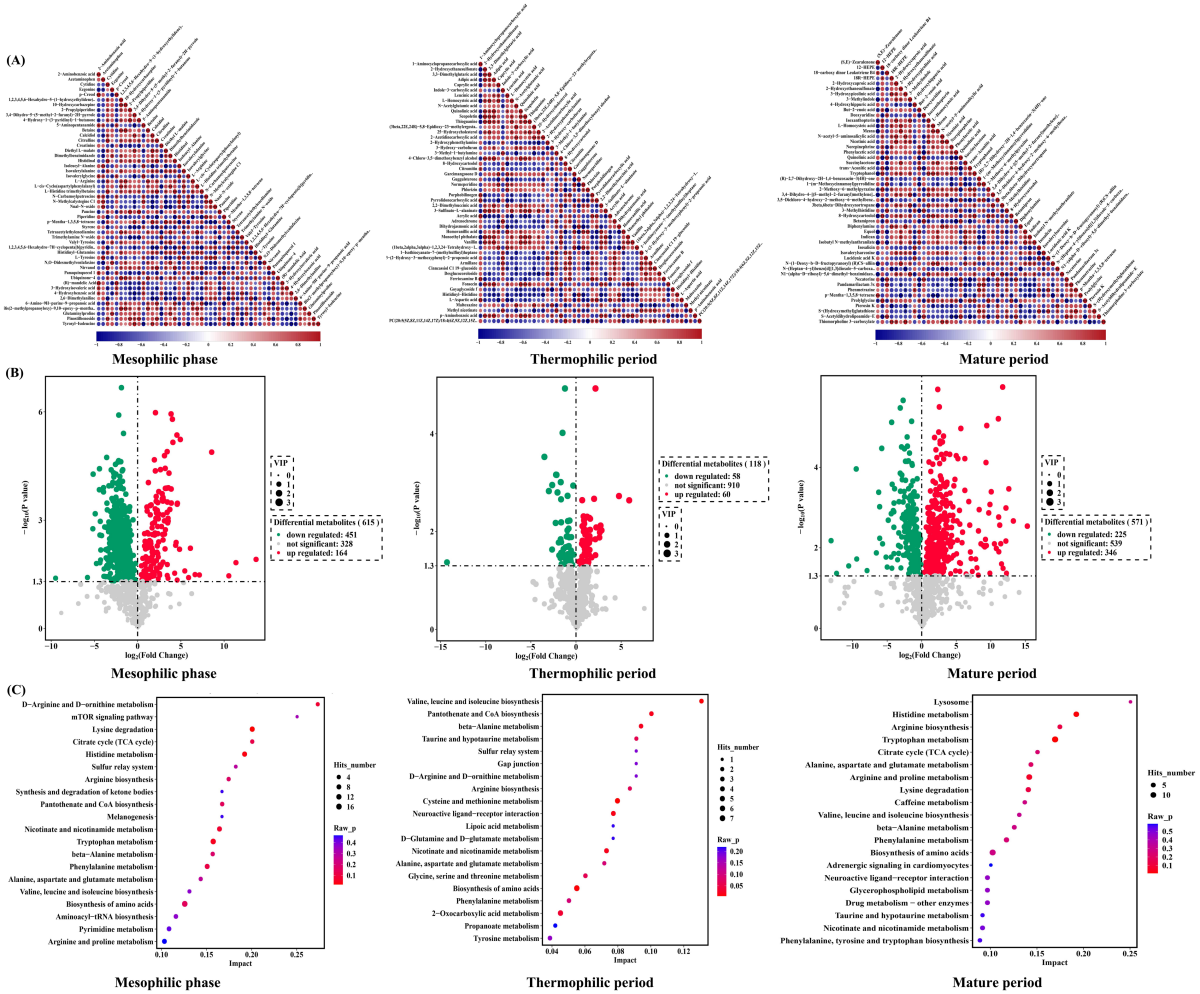
Distinct metabolome in chicken manure composting. **(A)** Correlation heat map of differential metabolites; **(B)** Volcano plot of differential metabolites; **(C)** Bubble plot of differential metabolic pathways.

A total of 1,304 metabolites were identified during chicken manure composting (Figure 2A). When comparing the metabolites between the inoculation and control groups, 615 distinct metabolites were detected during the mesophilic phase, among these, 164 metabolites were upregulated, while 451 were downregulated. During the thermophilic period, 118 distinct metabolites were enriched with 58 downregulated and 60 upregulated. In the mature phase, a total of 571 distinct metabolites were identified, with 346 upregulated and 225 downregulated (Figure 2B). Based on KEGG results, there were 71, 50, and 65 distinct metabolic pathways detected during the mesophilic, thermophilic, and mature periods, respectively (Figure 2C). Notably, alanine, aspartic acid, glutamic acid, and tryptophan metabolism pathways exhibited significant differences during the mesophilic period. The thermophilic period revealed key metabolic pathways, including the tricarboxylic acid cycle, alanine metabolism, histidine metabolism, linoleic acid metabolism, piperidine metabolism, pyrimidine metabolism, and amino acid biosynthesis. In the mature period, significant pathways included histidine metabolism, tryptophan metabolism, and arginine and proline metabolism.

### 3.3 Alpha diversity analysis

The microbial diversity of the inoculated and control groups was assessed across different periods (Table 1). Changes in the Chao1 index revealed that the control group exhibited higher values compared to the inoculated group. This index showed a decreasing trend with rising temperatures and an increase when temperatures decreased during the mature stage. The Good’s coverage for the inoculated group remained at 99% across all composting periods, while the control group demonstrated 99% coverage during the initial, mesophilic, and thermophilic periods and 98% during the mature phase. Both the Simpson index and Shannon index exhibited a negative correlation with temperature, decreasing as temperatures increased and rising as temperatures decreased, reaching their lowest values during the thermophilic period. Throughout the composting process, the mean values for both the Simpson and Shannon indices were higher in the control group compared to the inoculated group.

**Table 1:** Alpha diversity index statistics.

		Initial	Mesophilic	Thermophilic	Mature
Inoculation	Chao1	1187.37	1161.81	1089.68	1459.54
	Good’s coverage	99%	99%	99%	99%
	Simpson	0.96	0.93	0.89	0.92
	Shannon	5.68	5.2	5.31	5.76
Control	Chao1	1231.92	1207.48	1153.53	2080.96
	Good’s coverage	99%	99%	99%	98%
	Simpson	0.95	0.98	0.94	0.98
	Shannon	6.35	6.11	5.61	7.98

### 3.4 Microbial community analysis

**Figure 3:**
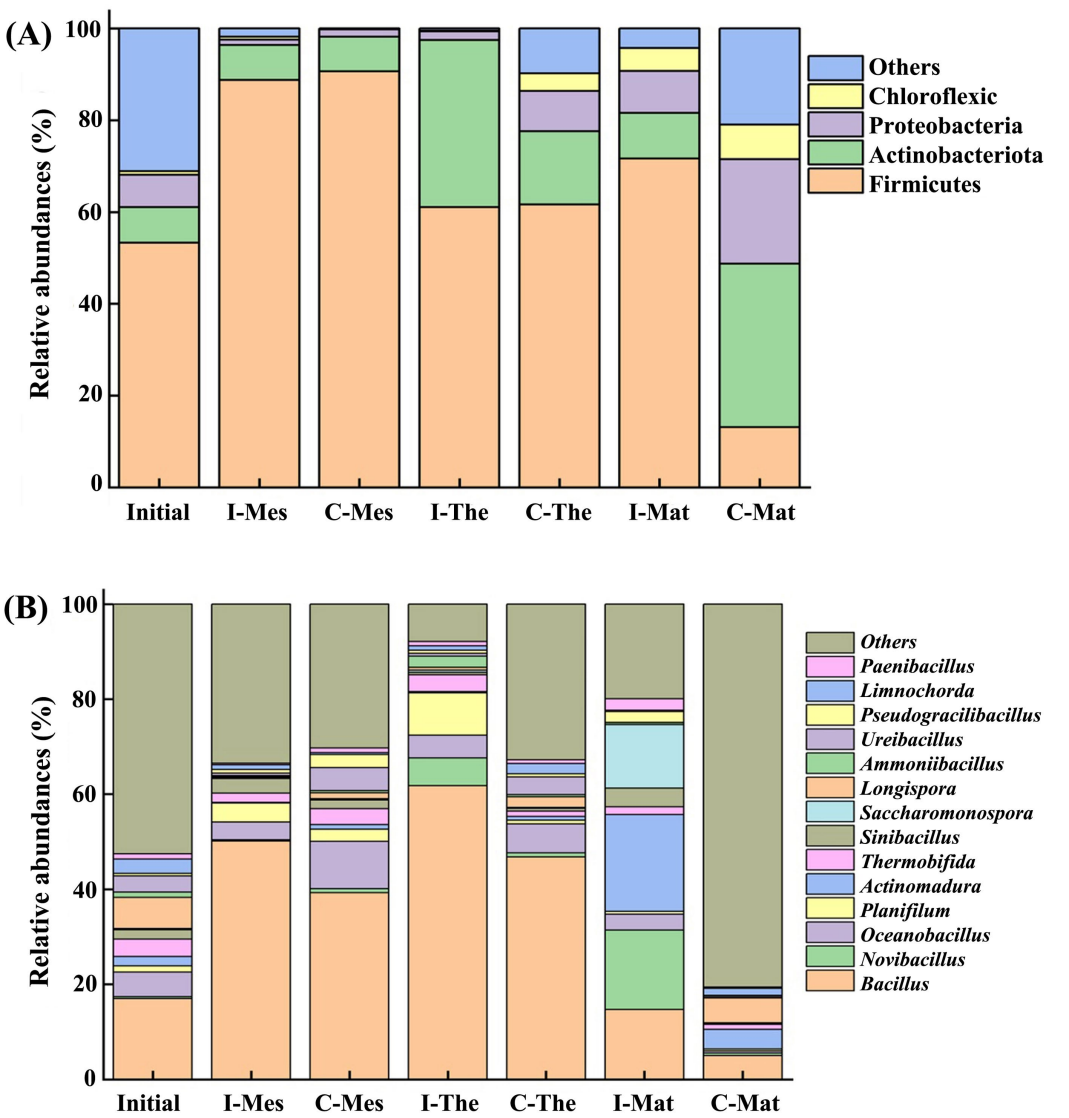
Relative abundances of bacterial species at phylum **(A)** and genus **(B)** levels in chicken manure composting. I, inoculation; C, control; Mes, mesophilic period; The, thermophilic period; Mat, mature period.

Analysis of changes in microbial community structure revealed that Firmicutes, Actinobacteria, Proteobacteria, and Chloroflexi were the most prominent phyla during the chicken manure composting periods, with average relative abundances of 62.86, 17.26, 7.48, and 2.59%, respectively (Figure 3A). Compared to the initial composting period, the relative abundance of Firmicutes increased, reaching 90.62% in the inoculation group and 71.64% in the control groups during the mesophilic period. By the end of the composting process, these relative abundances decreased to 61.07% in the inoculation group and 13.12% in the control group, likely due to a decrease in temperature. During the mesophilic period, the relative abundance of Proteobacteria decreased from 7.00 to 1.12%, showing no significant changes until the end of the composting process. However, during the maturation period, the relative abundance in the control group gradually increased from 9.20 to 22.77%. Additionally, the relative abundance of Chloroflexi also increased in the control group, while no significant changes occurred in the inoculation group, with final the abundances recorded at 7.55% for the control and 0.12% for the inoculation group.

At the genus level (Figure 3B), the dominant genera identified in chicken manure composting included *Bacillus* (36.28%), *Novibacillus* (4.19%), *Oceanobacillus* (4.72%), *Actinomadura* (4.13%), *Planifilum* (2.88%), *Saccharomonospora* (2.47%), *Thermobifida* (2.11%), and *Sinibacillus* (1.66%). The relative abundances of *Bacillus* and *Planifolium* initially increased and subsequently decreased in both compost groups. *Bacillus* exhibited a persistent increase from 17.03 to 61.77% during the thermophilic period, before declining to 14.70% during the mature period in the inoculation group. In the control, the relative abundance of *Bacillus* increased to 46.82% during the thermophilic period, then decreased to 5.01% by the mature period. The relative abundance of *Novibacillus* varied significantly between the two groups increasing from 0.35 to 16.27% by the end of the inoculation phase while remaining low at 0.55% in the control. The relative abundance of *Actinomadura* remained low before the thermophilic period and increased significantly from 1.98 to 20.33 and 4.13% in the inoculation and control during the mature period, respectively.

### 3.5 Relationship between physicochemical metabolisms and microbial structure

**Figure 4:**
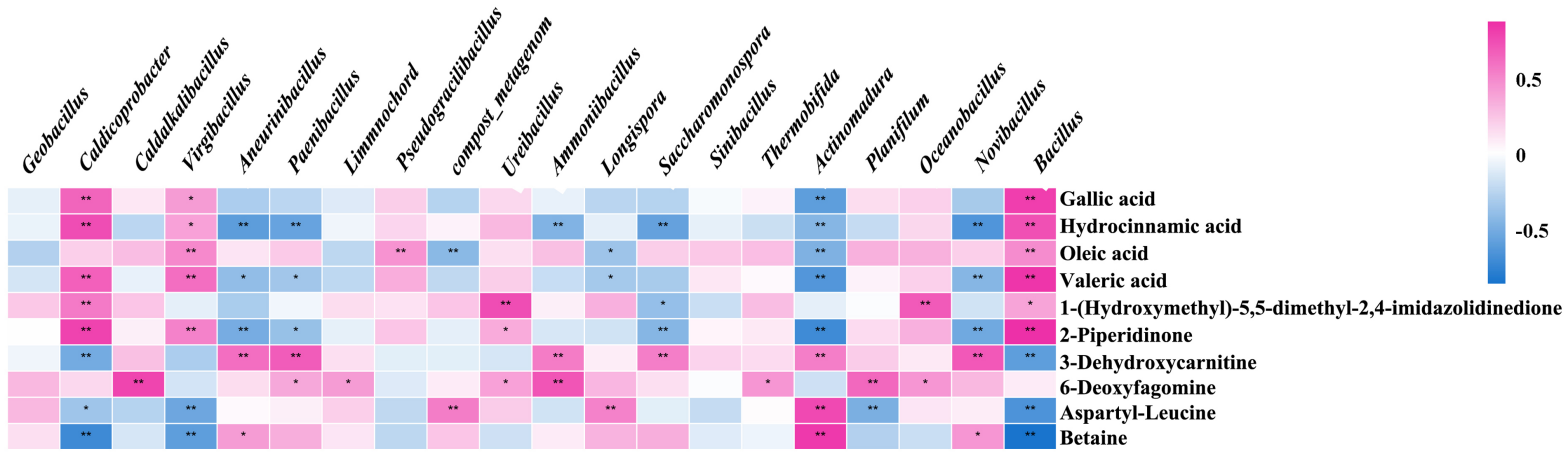
The relationship analysis of the main functional microbes and differential metabolites. The bacterial community and microbial metabolites are positively correlated in the red color, while the bacterial community and microbial metabolites are negatively correlated in the green color.

The relationship between the primary functional microbes and differential metabolites was analyzed. Gallic acid, hydrocinnamic acid, oleic acid, valeric acid, 2-piperidone, 3-dehydrocaritine, 6-deoxyrismamine, aspartyl-leucine, and betaine were the main metabolites related to microorganisms in chicken manure composting (Figure 4). During the composting period, gallic acid, hydrocinnamic acid, valeric acid, and 2-piperidone were enriched, showing positive correlations with *Bacillus*, *Caldicoprobacter*, and Virgibacillus, while demonstrating negative correlations with *Novibacillus*, *Actinomadura*, *Saccharomonospora*, *Paenibacillus*, and *Aneurinibacillus*. Conversely, the levels of aspartyl-leucine and betaine decreased during the composting periods, showing negative correlations with *Bacillus*, *Caldicoprobacter*, and *Virgibacillus*, and positive correlations with *Actinomadura*. Additionally, aspartyl-leucine was positively correlated with *Longispora* and the compost_metagenome, while betaine was positively correlated with *Aneurinibacillus* and *Novibacillus*.

### 3.6 Thermal stability analysis

**Figure 5:**
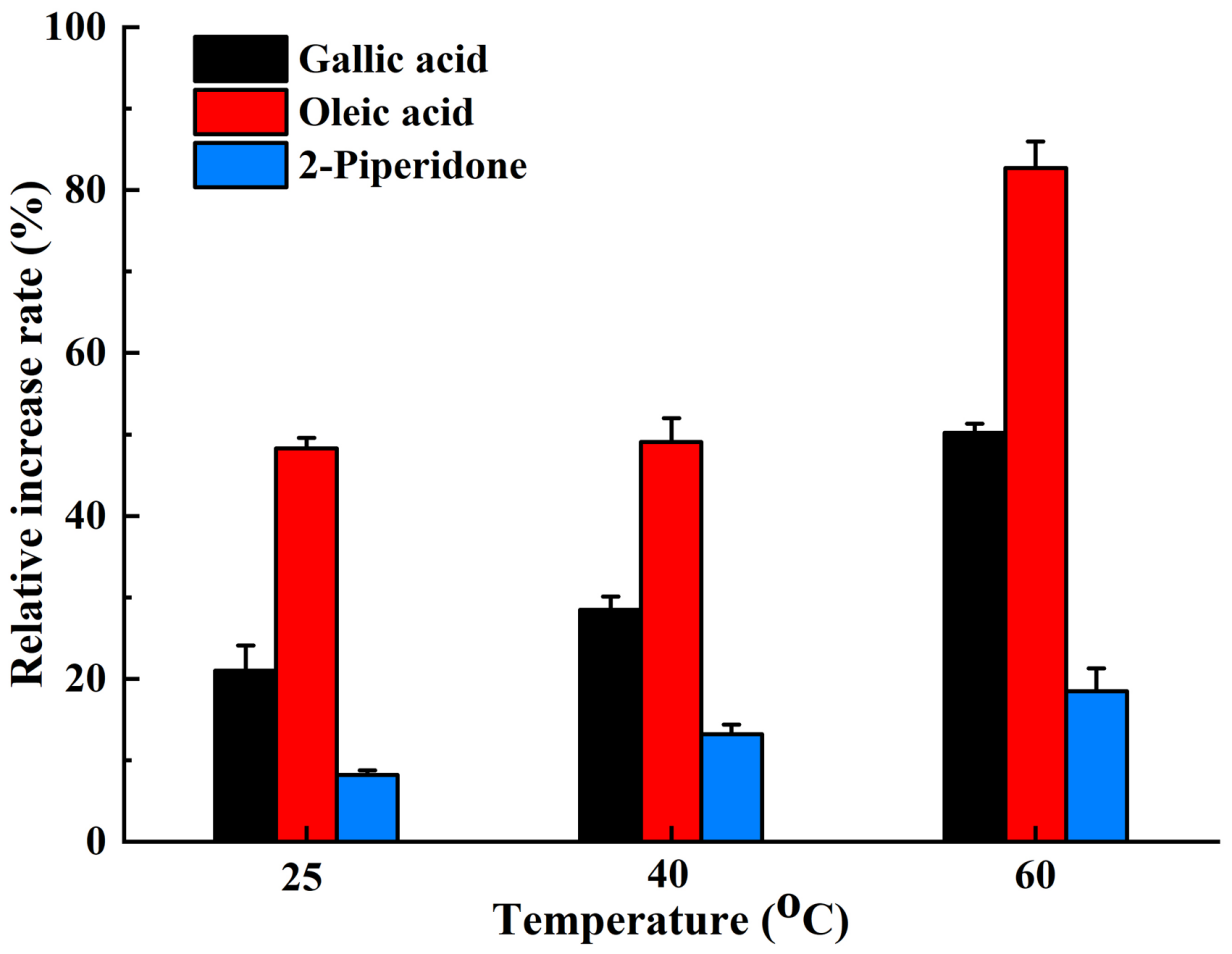
The thermal tolerance increase rate of the three heat stabilizers on the biocontrol agent at different temperatures. The data presented are the average values from triplicate technical repeats of measurements. The error bars represent the standard deviations of the means.

In the short-term thermal stability test of the compost biocontrol agent, the addition of three heat stabilizers—gallic acid, oleic acid, and 2-piperidone—significantly improved the survival rate of the biocontrol agent. This enhancement in vitality became more pronounced with increasing temperatures. The thermal stability of the biocontrol agent was insignificantly affected at 25°C and 40°C, but a marked increase was observed at 60°C. The bacterial colony count and growth status at this temperature were significantly better than those in the control group. Notably, the thermal stability of the biocontrol agent improved by approximately 50.22, 82.68, and 18.49% with the application of gallic acid, oleic acid, or 2-piperidone, respectively (*p* < 0.05), after being subjected to a temperature of 60°C for 20 h (Figure 5).

## 4 Discussion

Co-composting agricultural waste and livestock manure is an effective strategy for maximizing resource utilization (Wu et al., 2022). Mushroom residues exhibit contrasting characteristics to chicken manure, including lower moisture content, lower density, and a higher C/N ratio. This experiment demonstrates that incorporating mushroom residue as a cost-effective and efficient supplement for chicken manure composting can significantly enhance composting efficiency. A critical factor in assessing whether aerobic compost has successfully matured is temperature (Duan et al., 2019). Prior to this experiment, a composting test conducted exclusively with chicken manure revealed that the temperature did not rise significantly. Even with the addition of mushroom residue, the temperature consistently remained below 50°C, failing to reach the thermophilic phase. As a result, further analyses were considered unnecessary.

Through the co-composting of mushroom residues, chicken manure, and exogenous microbial agents, temperature trends resemble those observed in typical aerobic composting, characterized by three distinct phases: mesophilic, thermophilic, and maturation (Cao et al., 2020). In comparison to the control, the use of inoculum reduced the duration of the heating phase by 75% and elevated the peak temperature by 3.75 times. According to standards for effective composting, a thermophilic phase must last for more than 5 days (Jain et al., 2018). While several exogenous microbes, including *B. subtilis*, *E. hormaechei*, and *T. reesei*, have been shown to raise the temperature during the thermophilic phase by 3–7°C in chicken manure composting, this elevated temperature was only sustained above 70°C for a single day (Li et al., 2024). Maintaining elevated temperatures is critical for the effective elimination of pathogenic microorganisms and weed seeds in composting systems. In this experiment, temperatures exceeding 70°C were sustained for 30 days. This sustained heat may be attributed to the metabolism of specific ultra-high-temperature strains that rapidly decompose organic materials, thereby increasing temperatures. Additionally, these strains may synergize with the native microbial community, facilitating rapid heap heating and prolonging the thermophilic phase to enhance the composting process.

The degradation of a significant amount of organic matter in manure into organic acids results in a decrease in pH during the initial phase (Wang et al., 2019). As fermentation progresses, these organic acids are further degraded, and nitrogen-containing compounds undergo decomposition and ammoniation, leading to the synthesis of ammonium nitrogen, which significantly increases pH levels. The change in pH observed in the inoculated group was more pronounced due to the introduction of external microorganisms that enhanced organic matter degradation. Additionally, pH levels play a crucial role in microbial activity, thereby influencing compost quality. During the composting process, pH ranges from 3 to 11, with optimal fermentation occurring between 5 and 9, as most composting microbes thrive in neutral to acidic conditions (Farah et al., 2015). Thermophilic microorganisms secrete various extracellular enzymes that facilitate organic matter degradation during the thermophilic stage, subsequently mineralizing it into essential nutrients (Siles et al., 2016; Zhao et al., 2016). In contrast, the application of microbial inoculants enhances organic matter decomposition, resulting in significant increases in the nitrogen, phosphorus, and potassium contents of 3.61, 21.63, and 7.21%, respectively, (*p* < 0.05). Furthermore, soluble organic carbon and the total nutrient contents experienced substantial increases of 39.03 and 7.85%, respectively (*p* < 0.05). The organic matter content, which reflects microbial metabolism and nutrient conversion and release, is a crucial indicator of composting quality (Wang et al., 2019). Consequently, employing microbial inoculants during chicken manure composting can reduce gas emissions and mitigate nutrient losses.

During the thermophilic period, there were fewer distinct metabolic pathways, and the overall number of metabolites was lower compared to the mesophilic and mature periods. This trend is consistent with microbial diversity, which decreases during the thermophilic phase. Temperature significantly influences this decline, affecting microbial diversity and associated metabolic processes. Amino acid metabolism was observed throughout all three periods, likely due to the high nitrogen content of chicken manure. Notably, pathways involving the metabolism of alanine, aspartic acid, glutamic acid, and tryptophan were significantly different during the mesophilic period. The tricarboxylic acid cycle, alanine metabolism, histidine metabolism, linoleic acid metabolism, pyrimidine metabolism, and amino acid biosynthesis were among the significantly different metabolic pathways during the thermophilic period. During the mature period, the significantly different metabolic pathways were histidine metabolism, tryptophan metabolism, and arginine and proline metabolism.

Good’s coverage of all samples was over 98% (Table 1), indicating that the sequencing was adequate for all species in the samples (Qiu et al., 2022). During the initial to thermophilic period, the Chao1, Simpson, and Shannon indices decreased, suggesting a reduction in species abundance and diversity likely due to high temperatures inhibiting the growth of most bacteria. However, these indices increased during the mature period, which may be due to the reactivation of more microorganisms following a decrease in temperature. This phenomenon highlights temperature as a limiting factor in composting that may influence the formation of specific bacterial communities (Wei et al., 2018). Furthermore, the comparison showed that microbial diversity and richness in the inoculated group were significantly lower than in the control group, despite the inoculated group experiencing a longer warming rate and duration of high temperatures. This effect can be attributed to the inoculant used in this study, which was primarily composed of thermophilic bacteria capable of rapidly decomposing organic matter and generating heat. The increased proliferation of these bacteria within the compost system may inhibit the growth of other microorganisms, resulting in a decrease in overall microbial diversity and richness. Additionally, the thermophilic bacteria employed in chicken manure composting effectively break down the complex organic matter present in chicken manure, leading to a substantial rise in temperature within the compost.

Firmicutes emerged as the predominant bacterial group throughout the composting period. Due to their ability to produce spores, many Firmicutes bacteria are capable of surviving in extreme environments characterized by high temperatures and water shortages (Wei et al., 2018). These bacteria facilitate the hydrolysis of organic matter, including starch and protein, and are known to produce acetone and acetic acid, thereby contributing to a reduction in pH during the composting of chicken manure (Jin et al., 2022). The experiment observed that an increase in the abundance of Firmicutes and Actinobacteria led to the production of gallic acid and phenyl propionic acid, resulting in a decrease in pH during the degradation of organic matter. Additionally, Proteobacteria are adept at decomposing small molecular compounds such as glucose and butyrate, which supports the breakdown of amino acids and long-chain fatty acids, their relative abundance is influenced by the degradation of materials (Liu et al., 2022). Likewise, Chloroflexi play a role in the decomposition of organic carbon and endogenous humus, facilitating the breakdown of non-degradable organic matter within the materials (Fincker et al., 2020). The introduction of exogenous microbial inoculants resulted in a significant alteration in the microbial community structure, as evidenced by the differences observed between the inoculation and control groups within the compost. The inoculated group demonstrated increased levels of nitrogen (up 3.61%), phosphorus (up 21.63%), potassium (up 7.21%), soluble organic carbon (up 39.03%), and total nutrients (up 7.85%), indicating that the addition of exogenous microbial agents effectively promoted the degradation of organic matter. Notably, the control group retained a greater quantity of unprocessed material compared to the inoculated group, necessitating a more diverse array of microorganisms to efficiently decompose this raw matter in the later stages of composting (Meng et al., 2018). Moreover, the temperature was lower in the control group than in the inoculation group during composting process, which was suitable for the survival of a variety of non-functional microorganisms. This observation may clarify the higher abundance of Actinobacteria in the control group compared to the inoculated group during the thermophilic and maturation phases.

*Bacillus* serves as the primary functional bacteria for deodorization and cellulose degradation during manure composting (Wei et al., 2016). The relative abundance of *Bacillus* in both groups peaked during the thermophilic phase, with the inoculated group exhibiting higher levels compared to the control group throughout the composting process. This indicates that the decomposition efficiency in the inoculation group exceeds that of the control group. *Novibacillus*, belonging to the Thermoactinomycetidae family, demonstrates a strong capacity for the degradation of refractory azo substances at elevated temperatures, as it has been isolated from high-temperature composts (Yang et al., 2015; Tang et al., 2018). The data revealed a significant increase of 46.49 times in the relative abundance of *Novibacillus* in the inoculated group compared to the control group, where it remained consistently low. This disparity led to a lower efficiency in the decomposition of organic matter in the control group relative to the inoculated group. Another significant microbe, *Actinomadura*, also a member of the Thermoactinomycetidae family, displayed notable differences in relative abundance between the two groups, increasing by 10.27 times in the inoculated group and 2.08 times in the control group. This difference may be attributed to *Actinomadura*’s ability to promote cellulose degradation at high temperatures and enhance the degradation rate of chicken manure during composting through the secretion of extracellular thermostable xylanase (Wang et al., 2020; Taibi et al., 2012).

Based on the correlation analysis of the key functional microbes and metabolites with significant differences, the top 20 microbes were selected. These microbes are the predominant functional organisms influencing the metabolic processes during high-temperature composting. Interestingly, in the analysis of the relationship between main functional microbes and differential metabolites, *Bacillus*, *Caldicoprobacter*, and *Virgibacillus* were positively correlated with the majority of metabolites, while showing negative correlations with a few. Conversely, *Actinomadura*, *Saccharomonospora*, *Paenibacillus*, and *Aneurinibacillus* showed opposite correlations. *Bacillus*, being the primary bacterium for deodorization and cellulose breakdown in compost, reached peak relative abundance during the thermophilic phase across both groups, with the inoculated group consistently outpacing the control group. According to the KEGG pathway results, these metabolites are involved in amino acid, lipid, and endogenous metabolism.

Among the differential metabolites positively correlated with functional microorganisms during the thermophilic phase, 2-piperidinone has been shown to enhance the quality of organic fertilizers and stimulate plant growth (Qiu et al., 2019). Hydrocinnamic acid and oleic acid function as heat stabilizers, while gallic acid acts as a protectant against high temperatures. All these compounds demonstrate the ability to preserve cell vitality under elevated temperatures. The addition of 1.5 μM gallic acid enables chicken blood cells to withstand the oxidation of various components at high temperatures, thereby maintaining a high level of vitality (Srinontong et al., 2023). Moreover, when gallic acid was incorporated into vegetable oil, it remained unoxidized even at a high temperature of 180°C for 6 days (Jung and Choi, 2016). In the current study, a short-term thermal stability test was conducted on the compost biocontrol agent using three common thermostable compounds: gallic acid, oleic acid, and 2-piperidone. Changes in bacterial colony morphology indicated that the addition of heat stabilizers resulted in fuller colonies and improved the growth status of microorganisms. The count of viable bacteria indicated that the three selected heat stabilizers significantly enhanced the survival rate of the biocontrol agent at high temperatures. This vitality-enhancing effect became more pronounced as the temperature increased, aligning with previous research. The results also demonstrated that the secretion of specific metabolites by the composting inoculants serves as heat stabilizers, enhancing microbial activity and prolonging the duration of high temperatures. This capacity to mitigate the adverse effects of high-temperature environments on cells is crucial for sustaining the activity of key functional microorganisms, such as *Bacillus* and *Caldicoprobacter*, during the thermophilic phase. Consequently, their abundance increases, which enhances composting efficiency. This finding further substantiates the notion that the inoculated group exhibits higher decomposition efficiency than the control group. This complex interplay illustrates that microbial activity and material metabolism are interconnected during various composting stages, resulting in different metabolic pathways and functional microorganisms. This phenomenon may explain the dynamic correlation between material metabolism and functional microorganisms.

## 5 Conclusion

This study demonstrates that exogenous microbial agents can significantly enhance composting efficiency. Microorganisms modify the environment and metabolites through the decomposition of organic matter. The abundance and diversity of microbial flora are influenced by various metabolic substances. Analysis of the most critical metabolic pathways affecting the thermophilic stage suggests that elevated temperatures stimulate functional microorganisms to upregulate the expression of oleic acid and piperidine metabolic pathways. This leads to the production of oleic acid, gallic acid, 2-piperidone, and other thermal stabilizers and protectants. The effectiveness of these protective agents has been validated; with gallic acid, oleic acid, or 2-piperidone, the relative activity rates of the biocontrol agent increased by approximately 50.22, 82.68, and 18.49%, respectively, after exposure to a temperature of 60°C for 20 h. Consequently, this enhances the survival rate of microorganisms during the high-temperature phase and improves the catalytic efficiency of composting. The findings of this study provide a theoretical basis for further research on composting. This article investigates the transformations in the high-temperature composting process by examining the relationship between metabolites and functional microorganisms. However, this relationship may be complex and variable, involving multiple mechanisms that contribute to the progression of high-temperature composting. Therefore, further in-depth research in this area is warranted.

## Data Availability

The original contributions presented in the study are included in the article/supplementary material, further inquiries can be directed to the corresponding authors.
